# Haze in Beer: Its Formation and Alleviating Strategies, from a Protein–Polyphenol Complex Angle

**DOI:** 10.3390/foods10123114

**Published:** 2021-12-15

**Authors:** Yin Wang, Lingzhen Ye

**Affiliations:** 1 Institute of Rural Development, Zhejiang Academy of Agricultural Sciences, Hangzhou 310021, China; wangyin@zaas.ac.cn; 2 Zhongyuan Institute, Zhejiang University, Zhengzhou 450000, China; 3 Institute of Crop Science, Zhejiang University, Hangzhou 310058, China

**Keywords:** beer, haze, haze-active proteins, barley, molecular breeding, genes

## Abstract

Beer is one of the oldest and most widely consumed alcoholic beverages. Haze formation in beer is a serious quality problem, as it largely shortens the shelf life and flavor of beer. This paper reviews the factors affecting haze formation and strategies for reducing haze. Haze formation is mainly associated with specific chemical components in malt barley grains, such as proteins. The main factor causing haze formation is a cross-linking of haze active (HA) proteins and HA polyphenols. Many HA proteins and their editing genes or loci have been identified by proteomics and quantitative trait locus (QTL) analysis, respectively. Although some technical approaches have been available for reducing haze formation in beer, including silica and polyvinylpolypyrrolidone (PVPP) adsorbent treatments, the cost of beer production will increase and some flavor will be lost due to reduced relevant polyphenols and proteins. Therefore, breeding the malt barley cultivar with lower HA protein and/or HA polyphenols is the most efficient approach for controlling haze formation. Owing to the completion of barley whole genome sequencing and the rapid development of modern molecular breeding technology, several candidate genes controlling haze formation have been identified, providing a new solution for reducing beer haze.

## 1. Introduction

Beer is one of the oldest and also most widely consumed alcoholic beverages. It is a kind of colloid solution with complex composition and weak stability. The haze formation in beer is a serious quality problem, as it primarily affects the shelf life and flavor of beer. Hazes are caused by suspended insoluble particles of colloidal or larger size that can be perceived visually or by instruments.

**Figure 1:**
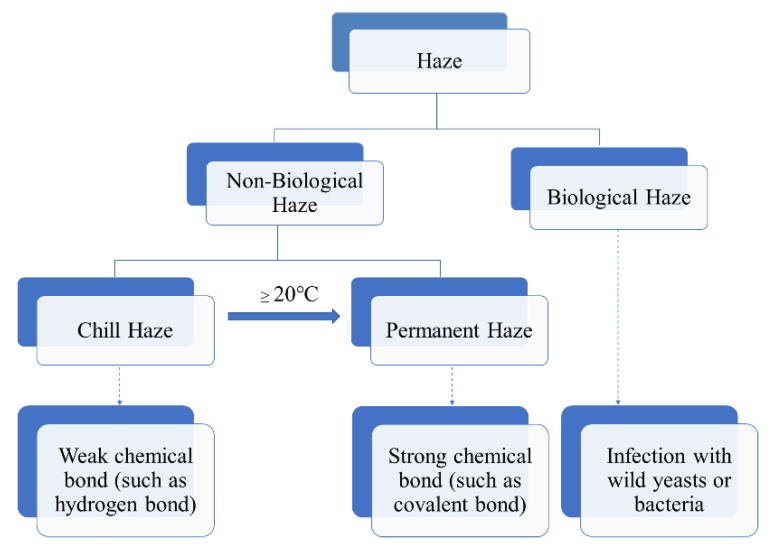
Scheme of haze formation in beer.

Beer haze can be divided into biological and non-biological ones. The biological haze can be reduced or avoided, as it is caused by wild bacteria or yeast due to poor hygiene during beer processing and storage (Figure 1). In contrast, the non-biological haze is difficult to deal with, is caused by the large molecular substances in beer, such as dextrin, β-glucan, proteins, and polyphenols, etc. [1,2,3]. In the process of storage and transportation, due to the light irradiation and vibration, the large molecular substances undergo changes such as combination and agglutination, resulting in the formation of turbidity. Therefore, the clarity stage of beer is temporary, whereas the final stage of beer is turbidity.

According to the European Brewery Convention (EBC), the non-biological haze was classified into two types: chill and permanent. The chill haze forms when the beer is chilled to 0 °C, but re-dissolves when the beer is warmed to 20 °C or more (Figure 1). The particle sizes existed in beer with chill haze range from 0.1 to 1.0 μm in diameter. While permanent haze is present in beer even at 20 °C or higher temperature, with particles ranging from 1 to 10 μm in diameter [4]. The small particles are more likely to be intermediate precursors for the larger haze particles [5]. It is generally believed that chill haze results from weak chemical bond (such as hydrogen bond) interaction between large molecular substances (such as proteins and polyphenols) in beer. While permanent haze is considered to be the result of strong chemical bonds (such as covalent bonds) interaction between the large molecular substances (Figure 1). In general, chill haze is a precursor of permanent haze, and the permanent haze is the product of the further oxidation polymerization of chill haze. Moreover, Mastanjevic et al., suggested metals support the transition of chill haze into the permanent one [6].

## 2. The Methodologies for Measurements of Haze in Beer

In the past, the turbidity of fluid (such as beer) was measured using a transparent container with a viewing panel attached to it, which could be viewed through the fluid. At present, some standardized analysis methods are used, including optical, microscopic, and enzymatic methods as well as particle size analysis. Moreover, these methods can also be used in combination [7]. Recently, Raman spectroscopy, especially TI-RMS (Turbidity Identification Raman Micro-Spectroscopy), was used to identify and differentiate turbidity particles in a complex solution such as beer. Although the analysis itself is fast and simple, the cost of the equipment is quite high; therefore, this evolving method is particularly recommended for large laboratories [8].

In general, the forced aging method is applied to predict the shelf life of beer by many beer companies. In detail, the turbidity of beer was tested after being stored for a specific time under certain conditions. The treatment condition and time were determined by the experimental purpose [4,9]. For example, Jongberg et al., used a forced aging method to investigate the haze formation in commercial beer [10]. The procedure is as follows: beer was exposed to aging treatment by heat/chill cycles of 60 °C for 48 h, followed by 0 °C for 24 h, according to the Analytica-EBC method 9.30 with slight modifications. Two levels were set as medium (samples subjected to five aging cycles) and high (samples subjected to ten forced aging cycles). However, the method is relatively time-consuming. A more rapid method, called the alcohol-chill test, is widely used. The procedures were as follows: 5% pure ethanol was added into beer sample and carefully mixed, frozen at −8 °C for 40 min, then measured by a turbimeter [11,12].

Many methodologies have been developed to determine the amount of haze active proteins in beer [13,14]. Currently, the method of tannic acid distribution droplets is most widely used, which is based on adding a fixed amount of tannin to an examined sample [15,16]. Due to the affinity between tannin and HA proteins, the turbidity formed during the aging process of beer. While the aging process of beer can be predicted by adding different concentrations of tannins. This method is characterized by the determination of tannin-related HA proteins. In addition, the saturated ammonium sulfate precipitation method is also used to determine the content of HA proteins [13,17].

## 3. The Reasons for Haze Formation in Beer

The stability of beer haze is primarily affected by the properties of malt barley, which is used as the main raw material for brewing. If the carbohydrates in malt are not sufficiently broken down during mashing, the long-chain dextrins cannot be used by yeast. After fermentation, dextrins will cause turbidity, as the solubility of dextrins is quite low in alcohol beverages [18]. Moreover, β-glucan in wort may increase turbidity due to its larger molecular weight [19,20]. Similarly, arabinoxylan is also a chemical compound causing beer turbidity through its connection with relevant proteins [21,22]. However, among the factors causing non-biological haze formation, the interaction between haze-active proteins and polyphenols is the most well-known [4,13,23].

### 3.1. Protein–Polyphenol Haze

**Figure 2:**
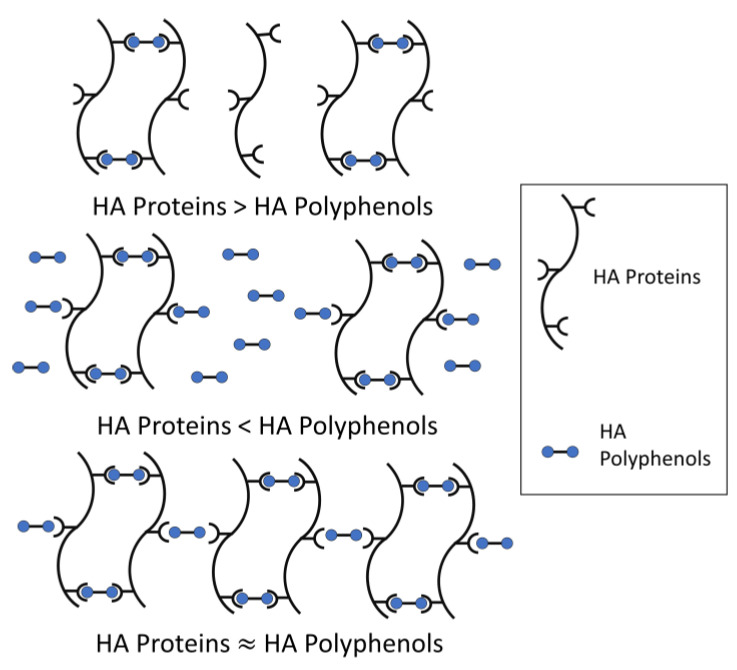
A model for protein–polyphenol interaction haze (modified from Siebert et al.) [23].

The model for haze formation of protein–polyphenol interaction has been described (Figure 2) [23]. According to the model, a haze active HA protein is conceptualized as having multiple binding sites with HA polyphenols, while a HA polyphenol has relatively fewer ends binding to HA protein, thus forming protein-polyphenol polymers with strong light scattering ability [24]. Meanwhile, beer haze stability depends on the proportion of HA proteins and polyphenols.

When HA proteins are relatively excessive, most HA polyphenols form dimers with HA proteins, and only a small amount of HA polyphenol binds to protein-polyphenol polymerization to produce a large cross-linking. Therefore, the formed particles are too small to form turbidity. Similarly, when HA polyphenols are relatively excessive, HA protein cannot provide enough binding sites for cross-linking and cannot form turbidity easily. Only in conditions where the total concentration of polyphenol ends is roughly equal to the number of binding sites in proteins, will turbidity be formed because of the development of such a large network, corresponding to large colloidal particles and maximum light scattering (Figure 2). Incipiently, the weak binding force between HA protein-polyphenol polymers is easy to re-break, which can be used to explain the formation of chill haze. Therefore, the initial reaction is more like hydrogen and/or hydrophobic bonding than covalent bonding, because most haze is caused by chilling partially or totally dissolves when a hazy beer is warmed. When these protein-polyphenol polymers are no longer broken, the reversible haze will become permanent [4].

### 3.2. Haze Active Proteins

Beer contains large amounts of barley proteins which have been hydrolyzed and chemically modified during malting and brewing processes. Leiper found that the mashing stage of brewing is most important in affecting the amount of protein in beer [21]. If the temperature of the protein decomposition stage is maintained at 48–52 °C, it may contain less total protein but more HA protein as the extra proteolysis. It was shown that as little as 2 mg/L of protein is sufficient to produce a haze of 1 EBC unit in beer [25]. Haze formation in beer is closely related to proteins derived from barley grains. A highly significant and positive correlation was found between haze characteristics and protein content in malt [26]. Moreover, the effect of barley protein on beer quality is also dependent on its components. Asano et al. believed that the main source of haze active proteins in beer was hordein [27]. Our previous studies showed that three kinds of proteins in barley grains (hordein, albumin, and globulin) were positively correlated with haze formation in beer, while hordein played the most important role [26]. Moreover, we found that the quantitative trait locus (QTL) controlling the tannin-related haze active proteins was located on a similar region of the gene encoding hordeins at 1H [16].

It was reported that the amino acid composition of HA proteins has a preference [21]. Several studies have suggested that proline and glutamine are important constituents of haze formation in beer [21,27,28]. The presumable binding site to HA polyphenol in HA proteins is proline residues [24]. However, free proline cannot combine with HA polyphenols to form turbidity [23]. The barley hordein is rich in proline (P) and glutamine (Q), and these two amino acids were usually in the adjacent state. It was assumed that such a P-Q-Q-P sequence was more suitable for HA polyphenol binding [27]. These proline-rich HA proteins are also found in apple juice [29] and grape wine [30]. Meanwhile, there are opposing viewpoints. Dadic et al., and Belleau et al., claimed that there is no special preference for the amino acid composition of HA proteins [31,32]. Iimure et al., found that proline content in the two identified haze proteins was only 6.6–8.7%, being much lower than that in the silica gel absorbing haze proteins [33]. We also studied the effect of the amino acid profile in malt on haze formation in beer [26]. It was found that proline, glutamic acid (glutamine), and phenylalanine content in malt were significantly and positively correlated with all haze parameters. Moreover, haze properties of the amino acid cannot be interpreted by hydrophilicity or hydrophobicity [26].

During beer filtration, silica gel is always used to remove HA proteins to reduce haze formation. The silica eluate (SE) proteins are mainly composed of hordein, being proline-rich and glutamate-rich [21,34]. Evans et al., and Robinson et al., suggested that the most attractive protein in SE proteins was barley trypsin inhibitor of the chloroform/methanol type (BTI-CMe) [34,35]. BTI-CMe belongs to the trypsin/a-amylase inhibitor family, and has a high abundance in barley endosperm [36]. We found BTI-CMe had 6 haplotypes by examining 37 barley accessions, and it was located on the short arm of chromosome 3H [37]. The polymorphism of the BTI-CMe band was detected in the different barley genotypes using immune-blot analysis with antibodies raised against SE protein. Some barley genotypes contain the band of 13.3 KDa (SE + ve), while others did not have the band (SE − ve) [34,37]. Moreover, by in vitro adding the synthesized BTI-CMe protein into commercial beer, we found that both original turbidity and alcohol chill haze degree of beer were increased. BTI-CMe of SE − ve haplotype showed a lower level of haze formation in beer than SE + ve haplotype. Furthermore, BTI-CMe has a significant interaction with tannic acid [38].

Currently, two-dimensional electrophoresis (2-DE) combined with mass spectrometry (MS) analysis has been widely used to study the protein composition in beer and beer turbidity. The relevant studies were summarized in Table 1. Obviously, the components of total proteins and HA proteins in beer are quite complex. However, the isobaric tags for relative and absolute quantification (iTRAQ) method, which is widely used in protein analysis of barley seedling, grain, and malt [39,40], is very limited in beer protein study. Therefore, a high-throughput method of protein identification should be applied in future studies.

In addition to the above-mentioned methods of reverse genetics (e.g., proteomics), a positive genetics strategy (e.g., QTL analysis) is also used to figure out the most important genes or proteins limiting beer shelf life [12]. There was a wide difference in the tannin-related HA proteins and chill haze stability among barley genotypes. The HA proteins and colloidal stability of beer are controlled by many loci. Furthermore, barley α-amylase/trypsin inhibitor CMb and CMd (BATI-CMb and BATI-CMd) were identified as two key genes controlling the chill haze stability of beer [12,16].

It is generally accepted that HA proteins have a higher level of proline [24]. The proline composition of proteins adsorbed onto silica gel was approximately 20 mol%, while those of BDAI-1, CMb, and CMe were lower than 10 mol%. Hence it is speculated that BDAI-1, CMb and CMe are not predominant haze active proteins, but they are initiation or growth factors in the formation of colloidal haze [33]. It may be concluded that HA proteins may consist of proline-poor proteins, such as BTI-CMe, BATI-CMd, and BATI-CMb, and proline-rich proteins such as hordeins.

### 3.3. Haze Active Polyphenols

Polyphenols in beer derive from both hops and malt. In detail, approximately 80% is originated from malt, while only 20% from hop-derived polyphenols [44,45]. It is well known that polyphenols play important roles in brewing [46]. Polyphenols are important flavor substances in beer, being closely associated with flavor and taste of beer. Polyphenols also greatly affect the colloid stability of beer, which can extend the shelf life of beer by acting as free radical scavengers and reducing agents [47,48]. For haze formation, the polyphenols are the potential substances that maintain the appropriate contents in beer. In this way, they ensure both the colloid stability and flavor requirement of beer. The most commonly found phenolic compounds in beer were catechin, epicatechin, Ferulic acid, p-coumaric acid, and vanillic acid [44]. These phenolic compounds can be easily transformed into highly flavor-active volatile, such as phenols 4-vinyl guaiacol and 4-vinyl phenol, which reduce beer flavor stability [46,49,50].

According to the molecular weight (MW), polyphenols in beer can be divided into tannic compounds (MW: 500~3000 U) and non-tannic compounds (MW < 500 U or MW > 3000 U). Phenolic compounds with a molecular weight less than 500 U mainly include phenolic acids, flavonols, flavanols, etc. Due to the excellent reducibility, these compounds were beneficial to the stability of beer. However, once these compounds are oxidized and polymerized, they will become the basis of beer turbidity. It was suggested that the complexed flavanols formed the bulk of the polyphenols in beer. McMurrough claimed that the low levels of such materials cause the problem of haze [51]. Polyphenols with molecular weight between 500 U and 3000 U can be simply called tannins, which are responsible for beer flavor and the main components of HA polyphenols. Polyphenols with molecular weights above 3000 U tend to precipitate easily, so most of them will be removed during brewing.

Because polyphenols are highly attractive to proteins containing proline residues [52,53]. They are major factors causing haze formation in beer, wine, and fruit juices [26]. The monomer polyphenols in beer tend to oxidize and polymerize, so that they have more ends that can bind to HA proteins, thus forming large HA polymers. The monomer polyphenols in beer mainly include flavonols and flavanols, with MW lower than 500 U. It has been found that the concentration of phenolic compounds with MW < 500 U was reduced gradually, as they were converted to tannins during beer storage [51]. Forced aging of beer experiments have shown significant losses of gallic acid, salicylic acid, hydroxy-phenyl lactic acid, chlorogenic acid, epicatechin, vanillic acid, ferulic acid, pyrocatechuic acid, and luteolin during the forced aging. It was supposed that these phenolic compounds took part in the colloidal changes, proposedly upon polymerization into tannins [10,54]. It has been reported that proanthocyanidins, one of the flavanol compounds, were important for the haze stability of beer. The haze stability of beer made from proanthocyanidins (PA)-free malt barley is better than that made from PA-containing barley [55].

## 4. The Passways for Preventing or Reducing Haze Development

According to the causes of beer haze formation, at present three strategies can be used to prevent or reduce haze development in beer: reducing HA polyphenols, reducing HA proteins, and removing both HA polyphenols and HA proteins at a certain proportion. These strategies can be implemented both in raw materials and during beer processing. To lengthen the shelf life of beer, the manufacturers now generally perform stabilizing treatments in beer processing. However, these treatments will increase the cost of beer production and also deteriorate some flavor due to reduced relevant polyphenols and proteins [56]. On the other hand, developing the malt barley cultivars with lower HA proteins and/or HA polyphenol is the most efficient way to control colloidal haze formation in beer.

### 4.1. Improving Beer Processing Technology

In beer production and processing, the common methods of improving colloidal stability include polyinylpolypyrrolidone (PVPP) adsorption, silica gel adsorption, tannic acid treatment, papain treatment, and so on. Tannic acid has a similar molecular structure as HA polyphenols. So, tannic acid will be a relatively specific precipitant of haze active proteins in beer [57]. The role of papain treatment is mainly that of breaking down of HA proteins to reduce beer haze formation [58]. However, some stabilizers, such as papain and tannin, are prohibited in some countries due to the purity requirement. In practice, breweries today mainly use PVPP or silica gel preparations. These two stabilizers can also be used in combination.

PVPP is widely used to extend the shelf life of beer, as it can adsorb both flavanoids and tannins [59]. It was reported that PVPP improves taste quality by removing harsh and astringent contributors. There is a disputation about its effect. O’Reilly suggested PVPP preferentially adsorb highly hydroxylated flavanoids promoting aging [60]. However, many research and practices have proved that PVPP can prolong the shelf life of many beverages [61,62]. The binding capacity of PVPP to polyphenols is about 90 mg polyphenol per 1 g PVPP [24,63]. PVPP is generally used at the secondary filtering stage in beer production. Specifically, the large particles of beer turbidities are firstly removed by silica gel adsorption. However, HA polyphenols still largely exist in beer. Then PVPP is applied to extend the shelf life of beer. Moreover, although PVPP can adsorb many HA polyphenols, it has little effect on turbid active proteins and foam active protein [29]. On the other hand, O’Reilly reported that PVPP is beneficial for foam stability [60].

The role of silica hydrogels is to absorb HA proteins in beer [21,24]. It is widely and conveniently applied in beer production as it can be processed into various specifications according to requirements, and the price is relatively low. It is claimed that equivalence of 1 g/hL PVPP is 7 g/hL silica hydrogel [60]. McKeown et al., reported that the most effective pore size for silica hydrogel is 3–12 nm [64]. Fernyhough et al., studied the development models of silica hydrogels in beer [65]. They found that application of silica at 75 g/hL could increase shelf life by 3–6 months, and double use of the silica prolongs the shelf time by double [65].

### 4.2. Improving Malt Barley Cultivars

Improvement of malt barley cultivars is the most economical and effective way of alleviating the haze formation in beer. It has been demonstrated that haze in beer can be reduced by optimizing the composition of HA polyphenols and HA proteins in barley malt. It was reported that PA-free barley variety Caminant showed slight haze, meeting the breweries quality specifications. The haze stability of unstabilized PA-free beers is excellent and it is possible to mix PA-free and traditional malt in a proportion of 1:1 to obtain beer with satisfactory haze stability without technical stabilization [66]. The PA-free gene was mapped to chromosome 3HL [67]. Pilot brewing trials and in vitro addition assays have shown that the use of SE − ve malt for brewing could improve haze stability in the resultant beer [34,35,38]. A survey of 219 international barley varieties identified 181 as SE + ve and 38 as SE − ve. The SE − ve barley varieties included well-known Barke, Bowman, Copeland, Haruna Nijo, Vigis, and so on [35]. Obviously, the beer colloid stability can be improved by the genetic improvement of barley varieties. At present, a gene-specific diagnostic molecular marker of SE protein has been developed for marker-assisted selective breeding [37]. More molecular markers for the genes controlling haze formation may be expected to be developed, thus promoting breeding for high-quality malting barley varieties.

In recent years, with the rapid development of barley genome and pan-genome information [68,69,70], the identification and analysis of the genes associated with malt quality is greatly accelerated. The acquisition of these critical genes lays a foundation for molecular breeding. The recent development and application of gene-editing technologies based on CRISPR-Cas9 have allowed crops’ targeted and precise genetic manipulation [71,72,73,74]. The improvement of crop yields, quality, and stress resistance can be achieved by knockout and/or activating one or several genes that confer undesirable traits. For example, amylose content and quality of rice grains were improved by CRISPR/Cas9 editing of a Waxy gene [75]. Compared to HA polyphenols, the studies on HA proteins are more extensively conducted. The synthesis of polyphenols is involved in many genes. Therefore, it is more feasible to manipulate the HA protein-controlled genes.

Currently, using the information of barley whole genome sequence, the genes controlling the known HA proteins could be identified. Table 2 summarizes the candidate genes potentially useful for further genetic improvement. The next step is to reveal the functions of these genes. Meanwhile, the relevant molecular markers are developed for marker assist breeding (MAS) and precision breeding based on Cas9 technology.

## 5. Conclusions

**Figure 3:**
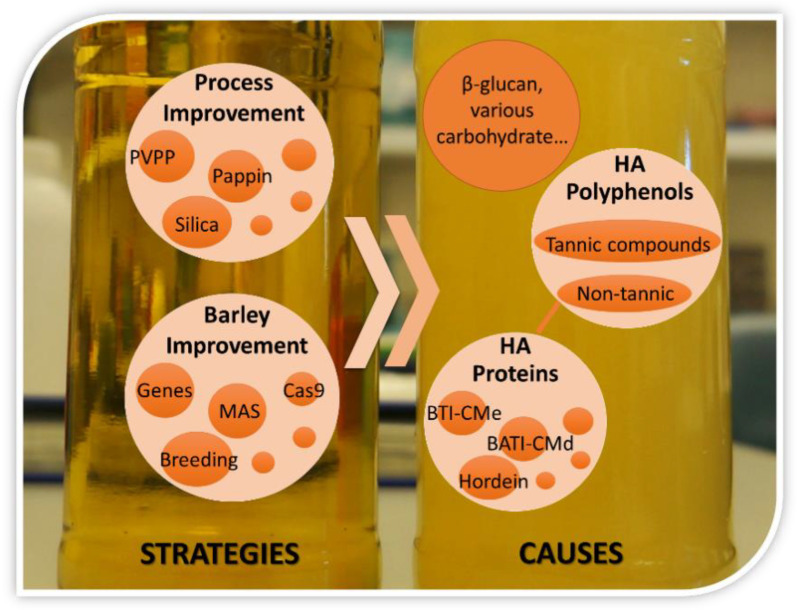
The diagram of causes and strategies for beer haze formation.

A summary is presented in Figure 3 for the reasons and alleviating strategies of haze formation in beer. Beer turbidity is dependent on many factors, but it is mainly affected by the quality of its raw material, barley malt. The haze formation in beer is closely related to the composition of starch, protein, polyphenols, and especially the interaction between HA proteins and HA polyphenol. Genetic improvement of some traits associated with haze formation is the most economical and efficient way to alleviate the haze in beer. With the development of rapid genomics and gene-editing technology, more genes controlling haze formation are being identified and used in malt barley breeding for improving malt quality.

## Figures and Tables

**Table 1:** Beer HA proteins identified by proteomics method.

Samples	Proteomics Methodology	Protein Species Resolved and Identified	References
Malt, Beer	Lab-on-a-Chip technique (capillary gel electrophoresis)	Protein fraction with a size of 25–28 kDa caused increased turbidity in the beer.	[21]
Barley, Silica adsorbed proteins	2-DE, combined with MS analysis followed by a database search, Western blot	SE protein (BTI-CMe)	[34,35]
Barley, Malt, Beer	2-DE, combined with MS analysis followed by a database search	A total of 40, 41, and 30 heat-stable water-soluble proteins were identified in barley, malt and beer, respectively, e.g., serpin-like chymotrypsin inhibitors (protein Z), amylase and amylase-protease inhibitors, and lipid transfer proteins (LTP1 and LTP2).	[41]
Beer haze samples, Silica adsorbed proteins	Same as above	Barley dimeric alpha-amylase inhibitor (BDAI-1), CMb component of tetrameric alpha-amylase inhibitor (CMb), Trypsin inhibitor CMe precursor (CMe), Protein Z4, Protein Z7, Trypsin/amylase inhibitor pUP13 (TAI)	[33]
Malt, Colloidal storage haze	Same as above	A total of 15 and 8 protein spots were identified as hordein in malt and beer turbidity, respectively	[42]
Fresh and old beers	Same as above	Protein Z4, LTP1, CMb, CMe, pUP13, 3a, and Bwiph were identified as constituents of the haze proteome.	[43]
Beer chill haze samples	PAGE, combined with MS analysis followed by a database search	BTI-CMe, BATI-CMd, BATI-CMb	[16]

**Table 2:** The candidate HA proteins and the corresponding genes in barley.

Candidate HA Protein	Accession Number	Corresponding Gene ID	Reference
Barley trypsin inhibitor CMe (BTI-CMe)	gi|19009; gi|1405736	HORVU3Hr1G013060; HORVU3Hr1G012970	[12,33,35,37,38]
CMb component of tetrameric α-amylase inhibitor (CMb)	gi|452323	HORVU4Hr1G081660	[12,33,43]
Protein Z4	gi|1310677	HORVU4Hr1G013480	[33,41,43]
Lipid transfer proteins 1 (LTP1)	gi|19037	HORVU5Hr1G046550	[41,43]
Lipid transfer proteins 2 (LTP2)	gi|19041	HORVU4Hr1G089500	[41,43]
Trypsin/amylase inhibitor pUP13	gi|225102	HORVU2Hr1G122280	[33,43]
Barley dimeric alpha-amylase inhibitor (BDAI-1)	gi|3367714	HORVU6Hr1G001150	[33]
Serpin-Z7	CAA64599	HORVU5Hr1G111920	[33]
Barley wound-induced protein homolog (Bwiph)	gi|256300	HORVU3Hr1G113120	[43]
D-hordein (fragment)	gi|671537	HORVU1Hr1G066650; HORVU1Hr1G064080	[43]
Barley α-amylase trypsin inhibitor CMd (BATI-CMd)	gi|585291	HORVU4Hr1G081640	[12]

## Data Availability

The data are contained within the article.
